# Making Research Matter: Comment on "Public Spending on Health Service and Policy Research in Canada, the United Kingdom, and the United States: A Modest Proposal"

**DOI:** 10.15171/ijhpm.2017.97

**Published:** 2017-08-13

**Authors:** David J. Hunter, John Frank

**Affiliations:** ^1^Institute of Health and Society, Newcastle University, Newcastle, UK.; ^2^Usher Institute of Population Health Sciences and Informatics, University of Edinburgh, Edinburgh, UK.

**Keywords:** Health Service and Policy Research (HSPR), Evidence, Health Reform

## Abstract

We offer a UK-based commentary on the recent "Perspective" published in IJHPM by Thakkar and Sullivan. We are sympathetic to the authors’ call for increased funding for health service and policy research (HSPR). However, we point out that increasing that investment – in any of the three countries they compare: Canada, the United States and the United Kingdom– will ipso facto not necessarily lead to any better use of research by health system decision-makers in these settings. We cite previous authors’ descriptions of the many factors that tend to make the worlds of researchers and decision-makers into "two solitudes." And we call for changes in the structure and funding of HSPR, particularly the incentives now in place for purely academic publishing, to tackle a widespread reality: most published research in HSPR, as in other applied fields of science, is never read or used by the vast majority of decision-makers, working out in the "real world."

The importance of, and need for, robust health service and policy research (HSPR) is well articulated by Thakkar and Sullivan in their perspective article.^1^ They claim that the funding devoted to HSPR is not in keeping with its value and importance although, certainly as far as the United Kingdom is concerned, there are early signs that this imbalance has not only been acknowledged but is being actively addressed, as we will demonstrate.

Strictly speaking, their comparison of “dollars spent” in 2010 on HSPR in Canada, the United States and the United Kingdom, is incorrect, in that it fails to adjust for the fact that virtually all Canadian research grants – unlike those in the United States and the United Kingdom – pay no portion of any investigator’s salary, and only a paltry overhead to their institution (less than 20% of direct costs, in comparison with much larger “overheads” paid in the United States and the United Kingdom – eg, 50% of the grant value in the United States’s NIH grants, including all investigators’ salary support). Based on the second author’s many years of grant writing and adjudication in all three countries, he reckons that these differences mean that a grant for research costing “x” dollars in Canada typically costs about twice that (ie, “2x” dollars) in the United States, and slightly more in the United Kingdom. Thus Thakkar and Sullivan’s estimate, that US$3.76 per capita was spent in Canada on HSPR in 2010, compared to US$6.46 per capita in the United States, would mean that about the same amount per capita was actually spent in the two countries on direct research costs, not including overhead and investigator salary support. [This has recently been pointed out by the Naylor Report on research policy and practice in Canada^2^ as a fundamental weakness of that system, in that research institutions effectively have to subsidize the full cost of doing research, putting them in a non-competitive position with research institutions in the United States and the United Kingdom.] However, we prefer to focus here on Thakkar and Sullivan’s main point: that HSPR funding could and should be more generous, in all three jurisdictions.

The authors refer to Berwick’s triple aim of improved population health, patient-centred care, and improved per capita care costs.^3^ This approach is at the centre of the significant transformation underway in the English National Health Service (NHS). Alongside the changes being implemented in regard to new models of care, there is significant investment in research to evaluate the impact of such changes and the accompanying barriers and facilitators, much of it funded by NHS England. The findings from these studies will be available shortly alongside a four-year national evaluation just commencing, funded by the National Institute for Health Research (the R&D wing of the English NHS). There seems to be no let-up in the appetite for more HSPR as far as the United Kingdom is concerned.

However, we suggest that funding is not the only, or even the most pressing, problem confronting HSPR. It is worth reminding ourselves that there already exists a modest evidence base in regard to health policy implementation and what needs to happen for successful transformation in health systems to occur. Yet, in the constant pursuit of new research, and at the risk of further stockpiling it, there are doubts concerning whether the extant body of research is being utilised to full effect. While we are not advocating a moratorium on new HSPR, unless it can be demonstrated that it is of value, especially at a time of fiscal constraint in public spending, then researchers could be leaving themselves vulnerable to challenge. Policy-makers among others are likely to call upon the research community to justify the existing investment in HSPR with potential implications for any significant increases in research regardless of how important and desirable it may be.

It is also important to explore the reasons for why HSPR has not, until recently, been viewed as a mainstream area for investment. As health systems globally seek to transform themselves, there is a growing recognition that evidence of what works and why (or why not) is urgently needed. Virtually all health systems are confronting the same challenges in regard to how to implement change successfully and sustainably.^4^ If, as we suggest, the existing evidence base is not being used to best effect we need to understand the reasons for such a state of affairs before investing significant new funding.

The reasons are multiple. Evidence often fails to impact on policy and practice because of failings among academic researchers whose findings take too long to produce, so that they miss the tide. Some of the reasons are beyond the power of the researcher to control and include delays arising from the bureaucratic and often painfully slow procedures displayed by ethics committees, research governance bodies and other hoops that have to be negotiated before any research can commence. Even when research studies are completed within a reasonable timeframe, the results are often perceived to be inaccessible and jargon-ridden and not presented in a format that engages busy managers and other health care professionals or gives them the information they need or are looking for. It is little wonder therefore that the output from think tanks and management consultants offers greater appeal because it is seen as timely and user-friendly — even if it lacks the academic rigour of sound, theory-driven, empirical research.

The incentive structure for academics is largely biased towards seeking publication of research in high-impact, peer-reviewed journals — rather than publishing in practitioner outlets which conceivably could have more impact on policy and practice. In the United Kingdom, the situation has begun to change with greater emphasis being placed upon impact. The last round of the Research Excellence Framework (the UK national competition for research financial support to universities, based on performance) in 2014 for the first time required the production of impact case studies to demonstrate the value in practice of funded research. While the shift to impact brings with it its own problems, in regard to spawning a new industry of case-study writers engaged in creative, and sometimes misleading, ways of demonstrating the value of research, the move has been generally welcomed. We therefore applaud the current effort to create epistemologically sound criteria for assessing whether a piece of research has had true societal impact while at the same time being mindful of a large existing literature which states that any one study should rarely be used as the basis for either policy or action, in that wherever possible replication is the essence of good science. Of course, we acknowledge in some complex contexts that may not be easy or always possible but it should be a goal to which researchers aspire.

In addition to the issue of impact, the way in which research proposals are designed, reviewed and implemented continues to place the emphasis upon, and privileges, academic-led research. This poses obstacles to providing useful, usable and timely research evidence in response to the needs of policy-makers and practitioners.^5^ While terms like co-production, co-creation and knowledge brokerage have entered the research vocabulary, their meaning often remains vague and imprecise and such fashionable approaches can seem tokenistic. Academics by and large continue to set the agenda and the systems and procedures for applying for and meting out research funding generally reinforce this bias.

But if HSPR is to have the impact it merits and is not going to disappoint end users, then those at whom the research is directed need to be involved at all stages of the research process from its inception through to dissemination of results.

HSPR is also difficult work and not always rewarding for academics accustomed to identifying clear cause and effect, and being able to definitively attribute change to particular interventions. By comparison with complex systems research, conducting systematic reviews of treatment effectiveness, and executing randomized controlled trials (RCTs) are straightforward and infinitely less problematic, in that the methods are well-codified globally. Conducting research in complex systems does not allow for such certainties or for tidy rational linear models to be adopted. Undertaking research in the messy real world where policies change with increasing rapidity, and “reform fatigue” and churn in the health system workforce are all too prevalent, add to the difficulties of conducting research to order. It is therefore hardly surprising that research in such contexts is not generalizable even if individual studies contribute to theory. Realist evaluation best captures the issues at stake here with its slogan, ‘what works for whom in what circumstances and why,’ being especially apposite.^6^

Working in a complex and often unpredictable environment requires not just adequate funding but the appropriate mix of skills. These are not just technical research skills around methods and design but include soft skills needed for relationship-building and exercising political astuteness. The temporal challenge has to be managed in order to balance the desire for real time research on the one hand with the need to conduct rigorous and sound research that is seen to be credible on the other.

The authors claim a connection exists between the performance of a country’s health system and investment in HSPR but the evidence offered in support of this relationship is negligible. Certainly the claim made for improvements in health system performance and quality under the UK Labour governments between 1997 and 2010 owed little if anything to research. The driver for reform was a politically mandated top-down management culture which adopted a ‘terror by targets’ regime. The Blair government was fixated on delivery and invested heavily in what has become known as ‘deliverology.’^7^ None of this had anything of significance to do with research. Indeed, much HSPR research on health systems change was ignored in the pursuit of an ideology-based agenda around privatisation, outsourcing and choice. Research critical of hospital mergers, for example, was ignored by a government which favoured them for largely political reasons. And research was consistently ignored that challenges the claims that opening up choice to patients improves care-system performance and quality of care.

An issue the paper does not deal with is the significant amount of in-house research that is conducted within health services but which is not included in the data on research funded through research councils and national programmes. Certainly in England, as noted earlier in regard to the current reforms underway, the NHS spends a significant amount on applied research undertaken by academics. Most of this research, which is sometimes categorised as consultancy, is not published and its impact on policy and practice remains unknown.

If there is to be greater stakeholder investment in HSPR, then those charged with producing it need to pay more attention to issues around impact and value for money. Attention needs to be given to ensuring that research already stockpiled is made available and appropriately presented to health service managers and professionals. Only then – after thorough review of what is already known — should new research be commissioned that adopts, and is committed to, co-production principles. That means negotiating with those at whom the research is targeted to ensure that the questions they want answered are addressed. And finally, the research needs to be carried out in a timely manner with regular feedback in order to ensure that as evidence emerges it can inform policy changes as they are evolving, with the potential for correction and amendment as required.

But it is not only researchers who need to up their game. Those who claim to want significant investment in HSPR and believe in its value need to be realistic in their expectations. Hard-pressed managers seeking quick wins in their efforts to transform services cannot expect favourable results to magically appear after a few months. For example, conducting health economics research on the cost-effectiveness of changes requires a significant longitudinal element in its design, to ensure sufficient confidence that there is a cost improvement over time.

Finally, in securing successful HSPR, attention needs to be given to the skills and capabilities of those conducting research. If the preference is for research that is co-produced with key stakeholders, to ensure the right questions are asked and to increase the likelihood of the research findings being owned and implemented, then having researchers equipped with the requisite soft skills alongside their technical academic skills is essential. Such skills include being able to build relationships, demonstrate political astuteness, communicate effectively with different audiences. Emotional intelligence remains rather uncommon among researchers, and yet is vital for research that seeks to inform and influence policy and practice. We know from the experiences of centres involved in public health research capacity-building in the United Kingdom that being aware of such factors and developing researchers accordingly is important and achieves results.^8^ Experiments with models such as the embedded researcher and researcher-in-residence that place researchers in policy and practice settings are demonstrating the value of bridging the academic research to policy and practice interface.^9^

## Ethical issues

Not applicable.

## Competing interests

Authors declare that they have no competing interests.

## Authors’ contributions

JF and DJH each contributed evenly to the conceptualization, writing and final editing of this commentary.

## Authors’ affiliations

^1^Institute of Health and Society, Newcastle University, Newcastle, UK. ^2^Usher Institute of Population Health Sciences and Informatics, University of Edinburgh, Edinburgh, UK.
